# A rare case of emphysematous cystitis and intravesical fungus balls caused by *Candida tropicalis*

**DOI:** 10.1186/s12879-026-14218-1

**Published:** 2026-08-31

**Authors:** Juanjuan Xie, Shifeng He, Xuan Zhang, Hao Chen, Hong Qian, Xuancai Chen, Yihui Liu, Hao Fu

**Affiliations:** 1 Department of Urology, The Affiliated Nanhua Hospital, University of South China, Hengyang, 421002 China; 2 Department of Pathology, The Affiliated Nanhua Hospital, University of South China, Hengyang, 421002 China

**Keywords:** Emphysematous cystitis, Urinary tract infection, *Candida tropicalis*, Fungus ball, Fungal infection

## Abstract

**Background:**

The concurrent fungal emphysematous cystitis (EC) and bladder fungus balls in patients with urinary tract infection is rare. Diagnosis and management remain challenging because of nonspecific clinical manifestations, limited imaging features, and the lack of standardized treatment strategies.

**Case presentation:**

A 62-year-old woman with poorly controlled type 2 diabetes mellitus (DM) presented with EC and bladder fungus balls caused by *Candida tropicalis*. Imaging revealed an intravesical mass surrounded by a large amount of gas. Conservative management, including antifungal therapy with fluconazole, insulin therapy, and continuous bladder irrigation with saline, was initially administered. However, persistent pain and intravesical gas required surgical intervention. Cystoscopy revealed multiple free-floating, oval, white fungal balls within the bladder, most measuring approximately 2.0 × 1.5 cm. Using a large-caliber endoscopic access sheath, transurethral cystoscopic irrigation was performed to fragment and evacuate the fungal balls. Postoperatively, the patient received systemic fluconazole therapy and bladder irrigation, resulting in recovery.

**Conclusion:**

The coexistence of fungal EC and bladder fungus balls should be considered in patients with DM and refractory or severe urinary tract infections when computed tomography (CT) demonstrates intravesical gas surrounding a bladder mass. Although systemic antifungal therapy, bladder irrigation, and glycemic control are primary treatment options, surgical intervention may be necessary when fungal balls are resistant to irrigation or when clinical improvement is not achieved. Transurethral cystoscopic irrigation using a large-diameter endoscopic access sheath appears to be an effective and safe treatment approach.

## Background

Fungal EC and bladder fungus balls are rare complications of fungal urinary tract infections (UTIs), although the prevalence of fungal UTIs has increased from 2% to 8% in recent decades [1, 2]. *Candida* species are the most common causative pathogens [3], and most reported cases involving concurrent EC and fungus balls have been associated with *Candida tropicalis* [1, 4]. Diagnosis is challenging because clinical manifestations are often nonspecific, with imaging findings playing a key diagnostic role [4]. Management remains non-standardized and depends on disease severity, ranging from antifungal and insulin therapy to bladder irrigation with saline or antifungal agents and surgical intervention [1, 4].

Here, we report the case of an older woman with EC and bladder fungus balls caused by *C. tropicalis*. The identified risk factors included poorly controlled diabetes mellitus (DM), and prior antibiotic exposure. Computed tomography (CT), urine culture, and cystoscopy confirmed the diagnosis. After conservative treatment with systemic antifungal therapy, bladder irrigation, and insulin therapy failed, transurethral removal of the fungus balls using a large-diameter endoscope access sheath, followed by postoperative fluconazole irrigation, achieved a favorable outcome. This report may provide useful insights for the diagnosis and management of similar cases.

## Case presentation

**Fig. 1:**
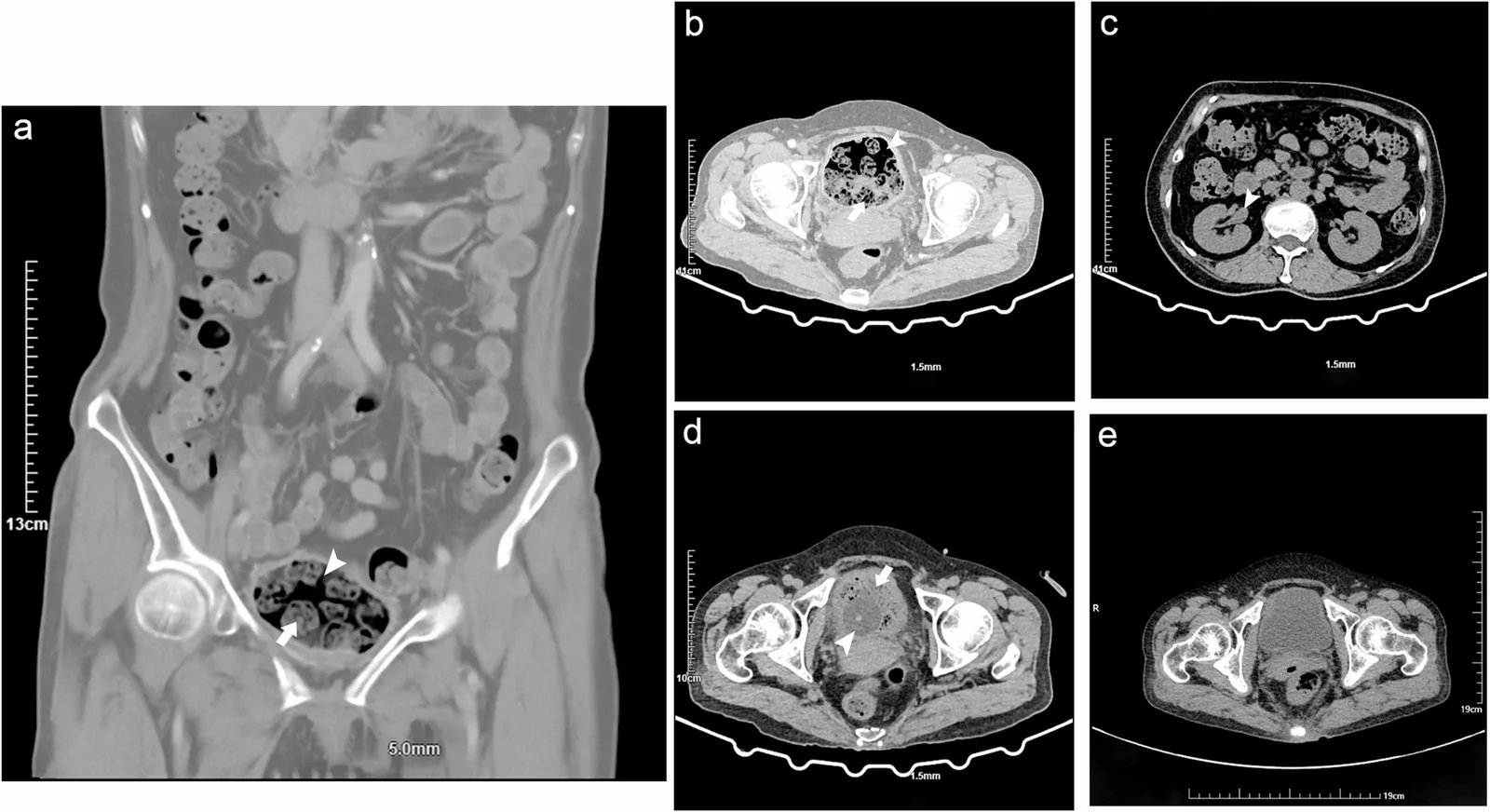
(**a**) Coronal view of abdominal CT demonstrating intraluminal gas (arrow head), multiples of isolated round masses (arrow) and bowel gas. (**b**) Axial contrast-enhanced abdominal CT scan (venous phase) showing a distended and clear outline bladder with diffuse wall thickening, irregular intraluminal gas (arrow), and intraluminal masses (arrowhead) similar to those described in a. (**c**) Mild right hydronephrosis. (**d**) Axial non-contrast abdominal CT scan after 1 week of antifungal therapy and bladder irrigation showing coalescent nodules (arrow) and the Foley catheter balloon (arrow head) within the bladder, with resolution of the previously observed gas. (**e**) Two weeks follow-up after discharge, pelvic CT demonstrated regression of the intramural gas and bladder mass

A 62-year-old woman with type 2 DM and poor adherence to insulin therapy was admitted to the urology department with a 4-week history of urinary frequency, urinary urgency, incontinence, gross hematuria, and lower abdominal pain. Before referral to our hospital, she had been diagnosed with atrophic vaginitis and UTI. Then she had received oral cefixime and intravaginal estrogen for one month. Physical examination revealed lower abdominal tenderness. Laboratory evaluation on admission revealed elevated blood glucose (15.69 mmol/L) and Hemoglobin A1c (HbA1c 10.5%), and elevated C-reactive protein (22.47 mg/L). Renal function was preserved, with serum creatinine (54 µmol/L) and Creatinine Clearance (52 mL/min). Urinalysis demonstrated severe hematuria (10,283 red blood cells/µL) and pyuria (2,250 white blood cells/µL), with a urine pH of 6.0. The ultrasound image showed no postvoid residual volume and no clinical evidence of bladder outlet obstruction such as stones, tumors and so on. Abdominal and pelvic enhanced (excretory phase) CT showed intraluminal gas, multiple isolated intravesical masses, diffuse-thickening and clear-outline of the bladder wall, gas in the intestinal tract without through the abdominal cavity and bladder lumen, as well as mild right hydronephrosis (Fig. 1a, b, c).

**Fig. 2:**
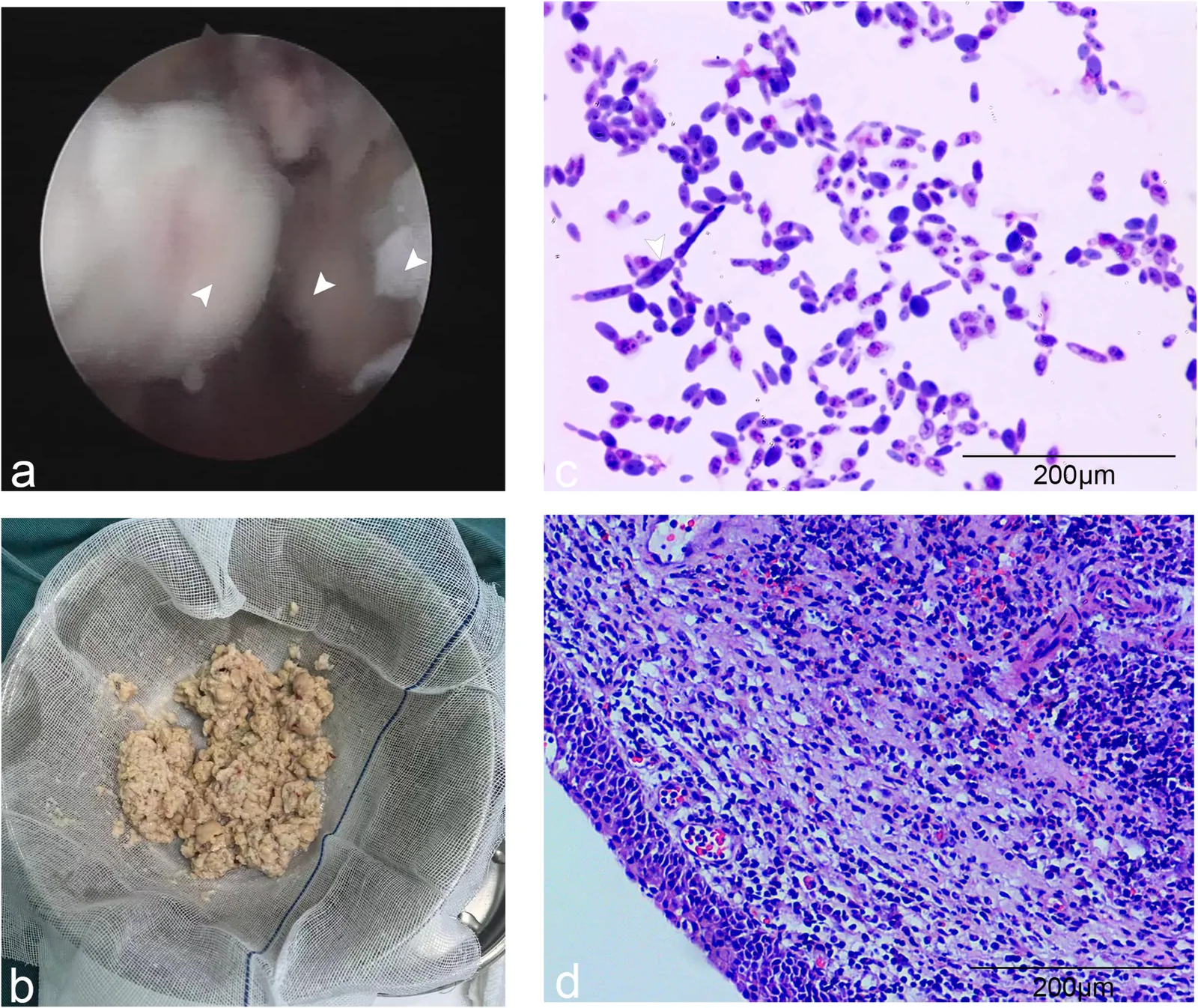
(**a**) Cystoscopy demonstrating multiple pale, oval, mobile, unattached masses measuring 1.5 × 2 cm in diameter within the bladder lumen (arrows), accompanied by bladder mucosal hyperemia. (**b**) Specimen obtained during transurethral removal showing grayish-whitish, friable, and soft bladder contents. (**c**) High-power view (×400) demonstrating numerous fungal hyphae (arrowhead) and spores with a characteristic moniliform appearance. (**d**) Histopathological section of the bladder mucosa demonstrating chronic suppurative inflammation (H&E stain, ×40)

Continuous irrigation via a three-cavity Foley catheter was initiated, which evacuated a large amount of gas and several flocculent materials. The patient was initially treated with ceftazidime, then escalated to piperacillin-tazobactam, and finally meropenem as symptoms progressed, along with insulin therapy for glycemic control. However, three consecutive bacterial urine cultures (two preoperatively and one postoperatively) yielded no bacterial growth. Two urine cultures obtained during hospitalization were positive for C. tropicalis (10⁵ CFU/mL), and blood cultures were also positive. Twice susceptibility testing showed the C. tropicalis isolate was susceptible to fluconazole and amphotericin B, but resistant to posaconazole. According to the susceptibility testing, intravenous fluconazole (Pfizer Inc.) was given at a single dose of 400 mg daily. After 1 week of intravenous fluconazole therapy, the patient continued to experience lower abdominal pain and pneumaturia. Cystoscopy revealed multiple whitish, oval, mobile, free-floating balls occupying the bladder lumen, most measuring approximately 1.5 × 2.0 cm (Fig. 2a). Axial non-contrast abdominal CT showed coalescent nodules within the bladder, with resolution of the previously observed gas (Fig. 1d). Surgical intervention was therefore considered necessary.

**Fig. 3:**
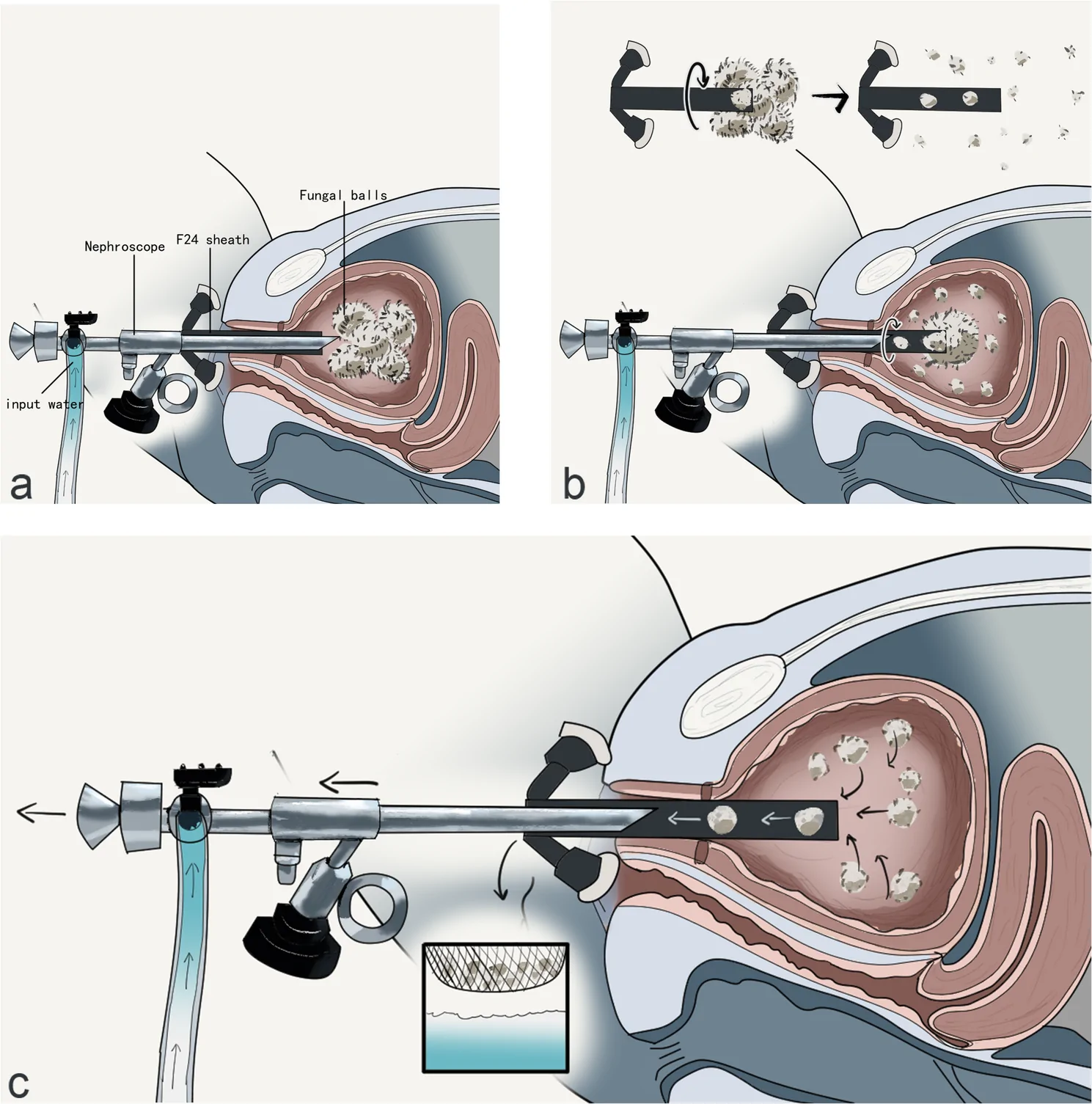
(**a**) A 24Fr disposable percutaneous renal endoscopic access sheath and the Nephroscope were introduced to the bladder lumen. (**b**) The sheath stabilized and scooped the fungal masses into little balls gradually. (**c**) These little balls were evacuated through the sheath under irrigation water flow

Under epidural anesthesia, a 24Fr disposable percutaneous renal endoscopic access sheath was introduced to the bladder lumen safely with a zebra guidewire. The scopy revealed that the bladder lumen was occupied by more than ten fungal balls distributed throughout the entire cavity, not attachment to the bladder wall. The bladder mucosa showed multiple scattered ulcerative lesions. Both ureteric orifices were clearly identifiable and uninvolved, with adequate bladder capacity and good visibility. With the guidance of a 8.5/11.5Fr Nephroscope (R.Wolf), the sheath stabilized and scooped the fungal masses into balls gradually, allowing the balls to be evacuated through the sheath by irrigation pressure of water flow at about 100 cmH_2_O (Fig. 3a, b, c). Finally, about 200 g whitish, soft fragments of the masses were retrieved (Fig. 2b). The operation was uneventful and lasted 70 min, without bleeding, bladder perforation and so on. Postoperatively, the patient received intravenous fluconazole at a dose of 400 mg daily for a week and daily intravesical fluconazole instillation (200 mg in 100 mL saline) retained 20 min for the first 3 days, and then continuous normal saline irrigation via a three-cavity Foley catheter for a week until a negative fungal urine culture was obtained. High-power view demonstrated numerous fungal hyphae and spores with a characteristic moniliform appearance (Fig. 2c). In Bladder biopsy demonstrated chronic suppurative mucosal inflammation (Fig. 2d). 

After 19 days of treatment in hospital, C-reactive protein levels and urinalysis findings had normalized. Glycemic control improved, and urine cultures were negative. The patient was discharged with an additional 2-week course of oral fluconazole (200 mg, daily). Four weeks follow-up after discharge, pelvic CT demonstrated regression of the intramural gas and bladder mass (Fig. 1e), as well as urine culture was negative. Long-term preventive measures included glycemic control, avoidance of unnecessary antibiotic use, and regular urological follow-up. The final diagnosis was EC and bladder fungus balls secondary to a *C. tropicalis* UTI.

## Discussion and conclusions

To our knowledge, the coexistence of fungal EC and bladder fungus balls in a patient with UTI is extremely rare, with this being only the third reported case since the first description in 1996 [1]. EC is a rare and potentially life-threatening infection characterized by gas within the bladder wall and lumen caused by gas-forming microorganisms [5]. The most common pathogens are members of the Enterobacteriaceae family, particularly *Escherichia coli* (60–70%) and *Klebsiella pneumoniae* (10–20%), whereas fungal pathogens such as *C. tropicalis* are uncommon [6]. Fungus balls, also termed fungomas or fungal bezoars, are aggregates of fungal hyphae mixed with cellular debris and urinary sediment [7]. Most affected patients are older women with poorly controlled DM and prior broad-spectrum antibiotic exposure, as observed in our case. Other risk factors include immunosuppression, indwelling urinary catheters, urinary tract obstruction, and neurogenic bladder [1, 8]. Topical vaginal estrogen preparations have been demonstrated to be effective in UTI of postmenopausal women because of the reestablishment of the normal lactobacillus-dominant vaginal flora [9]. However, Whether long-term intravaginal estrogen use contributes to fungal UTIs requires further investigation.

Diagnosis is challenging because symptoms are usually nonspecific and rely mainly on imaging, urine culture, and cystoscopy. Clinical manifestations range from mild urinary tract infection symptoms to abdominal pain and severe sepsis [1, 2, 10, 11]. Patients commonly present with urinary frequency, dysuria, gross hematuria, and lower abdominal pain, whereas pneumaturia is less frequently reported. Delayed diagnosis and treatment may result in serious complications, including bladder rupture and urosepsis [1, 10]. Imaging studies are essential, and all patients with DM and refractory UTI should undergo abdominal radiography and CT examination [1, 4, 12, 13]. Prompt urine and blood cultures are also necessary to direct antimicrobial therapy because some patients with EC may develop concomitant bacteremia (50%) or candidemia (1.3%) [2, 3]. Cystoscopy can directly identify fungus balls adherent to the bladder wall or floating within the lumen [4].

The treatment of EC and bladder fungus balls depends on disease severity and includes nonsurgical and surgical approaches. Conservative management is preferred initially and includes glycemic control, bladder drainage, systemic antibacterial and antifungal therapy for several weeks [3, 4, 13]. Intravesical instillation of fluconazole or amphotericin B may serve as an effective adjunctive therapy [3, 8]. However, surgical intervention may be required in patients who fail conservative treatment or develop severe necrotizing complications, including bladder rupture, peritonitis, or emphysematous pyelonephritis (EP) [3, 8]. Surgical options include suprapubic or transurethral debridement and, in severe cases, partial or total cystectomy [1, 4]. In patients with EP, prompt percutaneous renal drainage is necessary and may help avoid nephrectomy [6].

In our case, because the bladder was filled with a large number of fungal balls and necrotizing debris, early transurethral debridement was performed after initial medical therapy. We recommend the transurethral approach because the fungus balls were soft and could be effectively stabilized, fragmented, and irrigated using an Fr24 disposable percutaneous renal endoscope access sheath. Meanwhile, The Fr24 sheath was medium-soft medical polymers to avoid damaging bladder. The use of a large-diameter endoscopic access sheath during transurethral endoscopy maintained low intravesical pressure while facilitating efficient removal of fungal fragments.

However, the limitations of this case is that fungal hyphae were not demonstrated histologically using PAS or Gomori methenamine silver staining, and the removed intravesical fungal balls themselves were not examined with fungal stains. Because this report is a single case, further and long-term studies are needed to confirm the effectiveness and safety of this approach.

## Conclusion

Fungal EC with concomitant bladder fungus balls should be considered in patients with DM and refractory or severe UTIs when CT demonstrates intraluminal gas surrounding bladder masses. Systemic antifungal therapy, bladder irrigation, and glycemic control are the primary treatment options. However, surgical intervention may be necessary when fungus balls are resistant to irrigation or when clinical improvement is not achieved. The use of a large-diameter endoscopic access sheath combined with transurethral irrigation may be a feasible option.

## Data Availability

The datasets used and/or analysed during the current study are available from the corresponding author on reasonable request.

## Abbreviations

CT: Computed tomography
DM: Diabetes mellitus
EC: Emphysematous cystitis
EP: Emphysematous pyelonephritis
UTIs: Urinary tract infections

## References

[1] Comiter CV, McDonald M, Minton J, Yalla SV. Fungal bezoar and bladder rupture secondary to candida tropicalis. Urology. 1996;47(3):439–41. 10.1016/S0090-4295(99)80470-1 8633419

[2] Sahinler EB, Sinanoglu O, Erdogan E, Karaca Y. A Rare Cause of Urinary Retention Refractory to Conventional Measures: Bladder Fungoma. Cureus. 2022;14(11):e32024. 10.7759/cureus.32024 PMC9799711 36600849

[3] Kauffman CA. Diagnosis and management of fungal urinary tract infection. Infect Dis Clin North Am. 2014;28(1):61–74. 10.1016/j.idc.2013.09.004 24484575

[4] Wang L, Ji X, Sun GF, Qin YC, Gong MZ, Zhang JX, et al. Fungus ball and emphysematous cystitis secondary to Candida tropicalis: A case report. Can Urol Assoc J. 2015;9(9–10):E683–6. 10.5489/cuaj.3008 PMC4581947 26425243

[5] Nelson BA, Schouten WM. Emphysematous Cystitis. Mayo Clin Proc. 2021;96(6):1393. 10.1016/j.mayocp.2021.03.007 34088410

[6] Amano M, Shimizu T. Emphysematous cystitis: a review of the literature. Intern Med. 2014;53(2):79–82. 10.2169/internalmedicine.53.1121 24429444

[7] Albarrán Gómez MM, Molgado Garza VM, Cortés Becerra CM, González Garza R, Lozano Salinas JF, Gutiérrez González A, et al. Intravesical Fungus Ball Caused by Candida albicans in a Diabetic Patient With Septic Shock: A Case Report. Cureus. 2025;17(11):e97790. 10.7759/cureus.97790 PMC12740499 41458654

[8] Erkol Yilmaz Y, Havan M, Eyduran E, Penezoğlu N, Öcal D, Teber S, et al. Successful Treatment of Bladder Fungus Ball Due to Candida auris With Systemic/Local Amphotericin B and Surgical Excision. Case Rep Pediatr. 2025;2025:9741756. 10.1155/crpe/9741756 PMC12086029 40391003

[9] Schmiemann G, Kranz J, Mandraka F, Schubert S, Wagenlehner F, Gágyor I. The Diagnosis, Treatment, and Prevention of Recurrent Urinary Tract Infection. Dtsch Arztebl Int. 2024;121(11):373–82. 10.3238/arztebl.m2024.0068 PMC11539874 38686602

[10] Becker B, George A, Owen R. A nontraditional presentation and treatment for emphysematous cystitis. Urol Case Rep. 2023;46:102321. 10.1016/j.eucr.2023.102321 PMC9851856 36687746

[11] Liao PH, Hsieh MS, Chen YC. Emphysematous cystitis Cmaj. 2020;192(12):E313. 10.1503/cmaj.191288 PMC7101179 32392515

[12] Chew KKY, Kas M, Mancuso P. Urinary Tract Obstruction Secondary to Fungal Balls: A Systematic Review. Société Int d’Urologie J. 2024;5(3):227–36.

[13] Zhang L, Hong C. Urinary bladder obstruction caused by fungal balls: Unusual computed tomography findings - a case report. Med (Baltim). 2026;105(15):e48244. 10.1097/MD.0000000000048244 PMC13593163 41961699

